# Systematic review: predictive value of organoids in colorectal cancer

**DOI:** 10.1038/s41598-023-45297-8

**Published:** 2023-10-23

**Authors:** B. Cristoffer Sakshaug, Evelina Folkesson, Tonje Husby Haukaas, Torkild Visnes, Åsmund Flobak

**Affiliations:** 1 Department of Clinical and Molecular Medicine, Norwegian University of Science and Technology, Trondheim, Norway; 2 Department of Biotechnology and Nanomedicine, SINTEF Industry, Trondheim, Norway; 3 The Cancer Clinic, St Olav’s University Hospital, Prinsesse Kristinas Gate 1, 7030 Trondheim, Norway

**Keywords:** Chemotherapy, Radiotherapy, Targeted therapies, Colorectal cancer, Cancer models

## Abstract

While chemotherapy alone or in combination with radiotherapy and surgery are important modalities in the treatment of colorectal cancer, their widespread use is not paired with an abundance of diagnostic tools to match individual patients with the most effective standard-of-care chemo- or radiotherapy regimens. Patient-derived organoids are tumour-derived structures that have been shown to retain certain aspects of the tissue of origin. We present here a systematic review of studies that have tested the performance of patient derived organoids to predict the effect of anti-cancer therapies in colorectal cancer, for chemotherapies, targeted drugs, and radiation therapy, and we found overall a positive predictive value of 68% and a negative predictive value of 78% for organoid informed treatment, which outperforms response rates observed with empirically guided treatment selection.

## Introduction

Colorectal cancer (CRC) encompasses cancers of both colon and rectum. Data from the European Cancer Information System^1^ (ECIS) show that CRC is the second most common cancer and has the second highest mortality in the EU-27 countries. ECIS estimates that there are more than 300 000 new cases and more than 150 000 deaths from CRC every year in EU-27. Average 5-year survival from CRC in this area varies, but is on average slightly above 60% for CRC diagnosed from 2010 to 2014^2^. However, a large proportion of CRC patients present with metastatic disease, which has a substantially lower 5-year survival rate, at 14%^3^.

Treatment strategies for tumours eligible for curative therapy encompass all major cancer therapy modalities: radiotherapy, chemotherapy, and surgery. Radiation therapy is mainly used in rectal cancer. For colon cancer, chemotherapy is the main adjuvant treatment modality, and can be given both before and/or after surgery, depending on type and extent of disease. Surgery, however, remains the main curative treatment modality for localised colon and rectal cancer.

For advanced-stage disease, where treatment is not typically given with curative intention, chemotherapy is the most important treatment modality. 5-fluorouracil (5-FU), oxaliplatin, and irinotecan are the most commonly used drugs for CRC. They are usually given in one of three different combinations, namely FOLFOX (5-FU and oxaliplatin), FOLFIRI (5-FU and irinotecan), and FOLFOXIRI (5-FU, oxaliplatin and irinotecan). The same regimens are used when chemotherapy is given in a neoadjuvant or adjuvant setting. For rectal cancer, radiation therapy is often used in combination with chemotherapy in a neoadjuvant setting. In addition to this, some targeted therapies have been approved for use in advanced stage colorectal cancer.

Despite a growing arsenal of approved therapeutics, many patients will not experience effective first-line treatment with chemo- or radiotherapy, or even effective treatment at all^4^. Treatment strategies today are mainly decided empirically, as there are few biomarkers or tests available that reliably match patients with existing treatments. Where biomarkers for therapy selection exist, such as the use of genetic biomarkers to decide on cetuximab therapy in *KRAS* wild type tumours, these only have modest predictive value^5^. This means that many patients are exposed to the side-effects of anti-cancer therapies without receiving any of the benefits, and possibly even experiencing progression of disease while on ineffective treatment. In essence, there is a need for better ways of matching patients with effective anti-cancer therapies.

Traditional models of cancers, such as cancer cell lines and xenografted animal models are invaluable tools in cancer research, but neither of these models can bridge the gap between diagnostics and the clinic. Cell lines are inherently homogeneous and not able to accurately represent individual disease, and while xenografted animal models can be derived from patient tumours and as such hoped to faithfully represent individual disease, the process is ethically challenged, and far too time- and resource consuming to be scaled to the level that is needed to be a part of routine clinical decision support^6^.

A solution that has gained traction is the preclinical testing of chemo- and radiotherapies on patient-derived organoids (PDOs). These are three-dimensional cellular structures that can be cultivated and expanded in vitro from patient-derived material. They can be generated within a clinically relevant timeframe from most solid tissues, both healthy and cancerous, and have been shown to accurately recapitulate characteristics of these tissues genetically, transcriptionally, and in terms of histological architecture. Despite the use of different growth and cultivation protocols, these conclusions have been reached by several research groups^7–9^.

**Figure 1:**
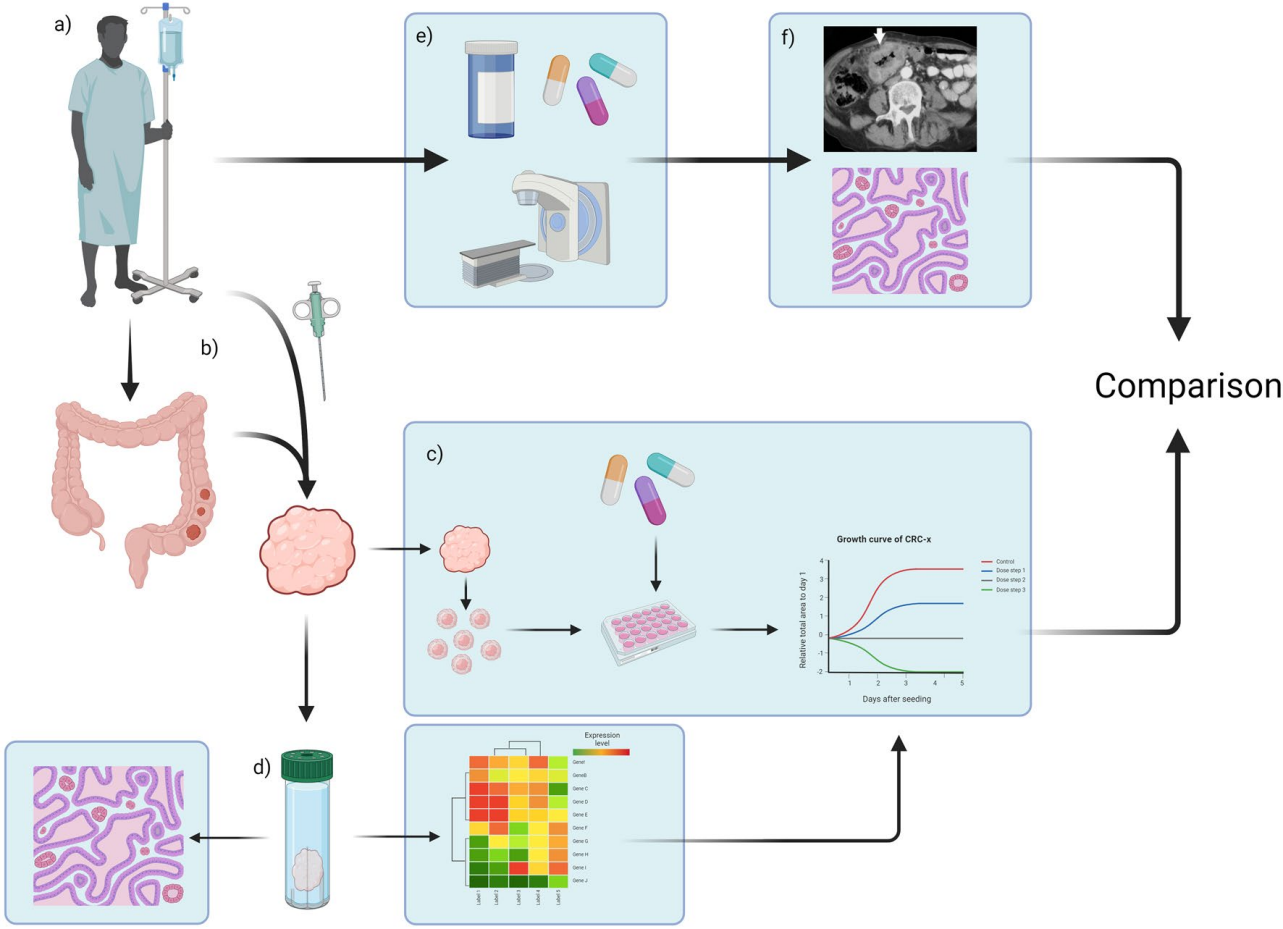
Example of study design in included studies. Steps a-d and e–f are conducted in parallel. (**a**) An eligible patient is selected and included in the study. (**b**) Tumour material is harvested, either via surgically resected material, or through needle-biopsies. (**c**) Tumours are processed into organoids and seeded with appropriate culture medium. Organoids are then exposed to the treatments of interest, and their response evaluated. (**d**) Some tumour material is set aside for further analyses, including histological and genetic analyses. (**e**) The patient receives appropriate anti-cancer treatment and (**f**) their response to the assigned therapy is evaluated.

Notably, over the past 3 years, several studies that compare the treatment responses of organoids to those of the patients from whom they were derived have emerged^10–29^. A visual representation of the typical design of these studies can be seen in Fig. 1. This systematic review is an attempt to summarize the findings of such studies that focus on CRC, and answer the question: *“Can cancer organoids predict treatment outcome for anti-cancer therapies in CRC?”*.

## Methods

### Search methodology

**Figure 2:**
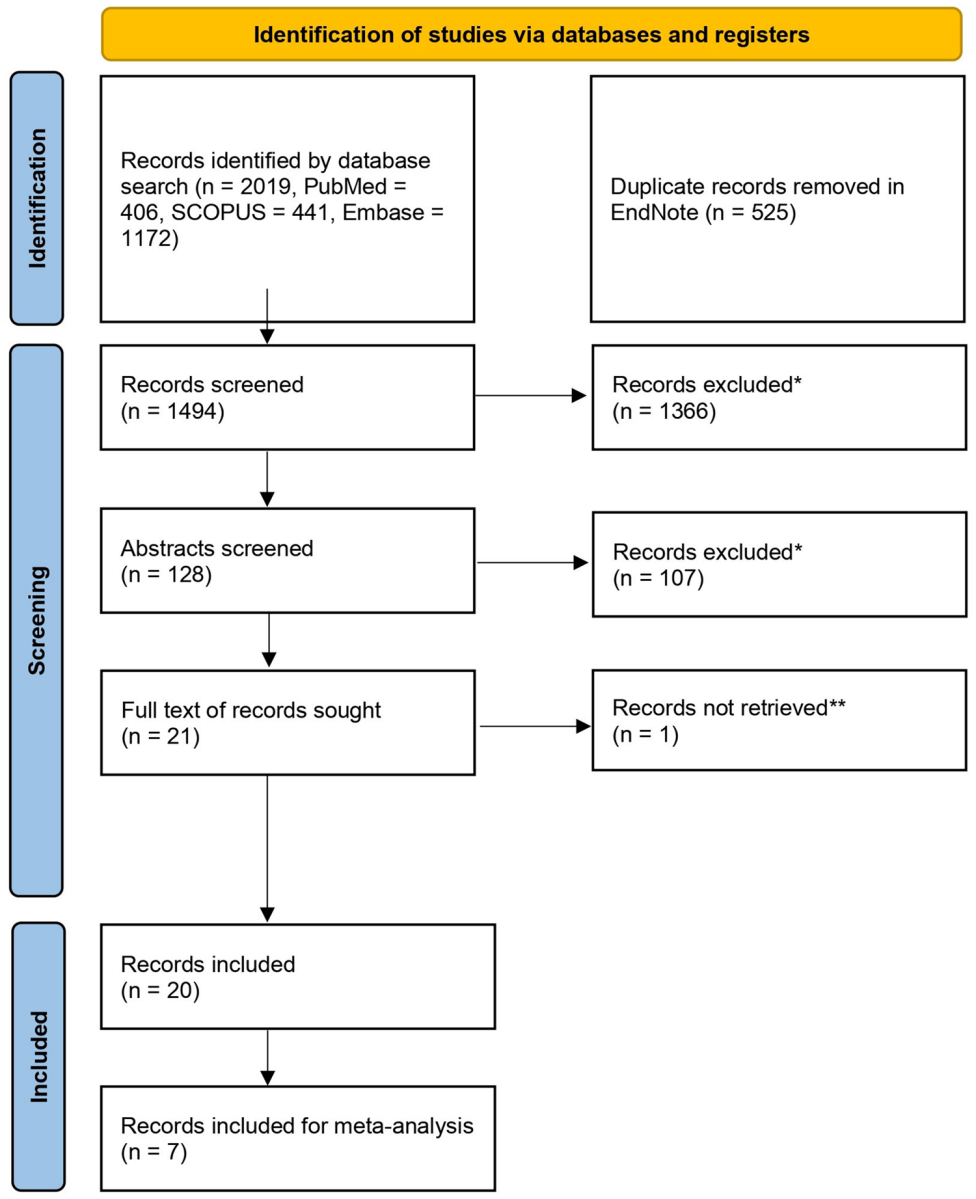
PRISMA-flow-diagram^30^ of record selection process. *Studies excluded were not compliant with the exclusion/inclusion criteria listed in Table 1. **Records not retrieved were not made available to us by the authors before submission.

The three databases searched were Medline, Embase, and SCOPUS. The first search was conducted in Medline via PubMed. This database was also used to identify effective keywords, based on some of the already known articles that inspired the review question^20,25^. Details regarding search terms and concept can be found in Supplementary Tables S1–S4. The search was last updated on the 14th of August 2023. For a detailed description of the search methodology, we refer to the Supplementary Information. Inclusion criteria for the selected studies can be found in Table 1*.* The study selection process is visualised in Fig. 2.

**Table 1:** Tabular overview of inclusion and exclusion criteria for the literature selection to support this review.

Inclusion criteria	Exclusion criteria
On topic with literature search	Off topic with literature search
Primary research article	Uses non-patient derived material for organoid formation (e.g., cell lines)
Uses patient derived material for organoid formation	Does not directly compare organoid and patient response to treatment
Compares organoid and patient response to treatment	Focuses on other cancers
Focuses on colorectal cancer

### Inclusion and exclusion criteria

#### Critical appraisal methodology

To our knowledge, there are no validated tools for the critical appraisal of cell-based studies. We therefore took inspiration from other sources, as well as from our prior knowledge of the field, to compose a list of criteria for the critical appraisal of the included studies. Specifically, inspiration was taken from the 2021 review from Verduin et al.^31^, and from general evaluation tools^32^. The criteria were defined to address three main categories: *reproducibility, translational potential,* and the *validity* of the results.

*Reproducibility* was addressed through the two first criteria in Table 2 (*detailed description of methods, data availability*)*.* There is no consensus on the best method for cultivating organoids, how samples should be exposed to therapy, which readouts should be used, or what threshold should be employed to classify a sample as sensitive or resistant to therapy. A detailed description of the methods used is therefore essential for the reproducibility of the study, and the data should also be readily available for critical assessment.

**Table 2:** Critical appraisal of included studies.

Study	Reproducibility		Translational potential		Validity	
	Detailed description of methods	Raw data available	Organoid culture success rate	Use of clinically relevant therapies	Number of patients included in response prediction	Classifier for organoid sensitivity
Vlachiogannis et al.^25^	Yes	Yes	70%	Yes	21	No
Ganesh et al.^13^	Yes	Yes	77%	Yes	26	No
Ooft et al.^20^	Yes	Yes	63%	Yes	29	Yes
Pasch et al.^23^	Yes	Yes	76%	Yes	1	Yes
Yao et al.^29^	Yes	Yes	85.7%	Yes	80	Yes
Janakiraman et al.^15^	Yes	Yes	90%	Yes	9	No
Narashiman et al.^19^	Yes	Yes	68%	Yes	2	No
Xu et al.^28^	Yes	Yes	100%	Yes	12	No
Wang et al.^26^	No	No	69.77% in cohort 1 and 80.21% in cohort 2	Yes	45	Yes
Ooft et al.^21^	Yes	Yes	57%	No	6	Yes
Park et al.^22^	Yes	Yes	70%	Yes	33	Yes
Cui et al.^11^	Yes	Yes	n.a	Yes	3	No
Cho et al.^10^	Yes	Yes	75%	Yes	40	Yes
Ding et al.^12^	Yes	Yes	100%	Yes	8	Yes, unspecified
Hsu et al.^14^	Yes	Yes	n.a	Yes	16	No
Mo et al.^18^	Yes	Yes	80.6%	Yes	23	No
Lv et al.^16^	Yes	Yes	88%	Yes	107	Yes
Tang et al.^24^	Yes	Yes	78.3%	Yes	113	Yes
Martini et al.^17^	Yes	No	80% in cohort 1, 60% in cohort 2	Yes	2	No
Wang et al.^27^	Yes	No	79.41%	Yes	108	Yes

Layout inspired by Verduin et al.^31^.

The *translational potential* was addressed through criteria three and four in Table 2 (*organoid success rate, use of clinically relevant therapies*). If organoids are not established successfully, it would not be practically possible to use them to guide treatment decisions. The use of clinically relevant drugs (e.g., standard of care chemotherapies, or approved targeted therapies) will also increase the translational potential of the study. Most importantly, the correlation of organoid response to patient response is necessary for the clinical implementation of organoids as predictors of treatment efficacy. This is addressed in the inclusion criteria.

The *validity* of the results was addressed through criteria five and six in Table 2 (*number of patients included, use/validation of classifier for sensitivity*). A high number of included samples/patients results in more robust evidence. The use and validation of a classifier for treatment sensitivity reduces the risk of confirmation bias, while simultaneously increasing the translational potential of the study.

### Meta-analysis

Seven of the included studies provided measures of accuracy, sensitivity, specificity, positive predictive value (PPV), negative predictive value (NPV), or the values needed to calculate these, and were thus eligible for our meta-analysis^12,16,20,24–26,29^. Four additional studies likely had these data but did not disclose them in the main text or the supplementary material^10,18,22,27^. The authors were contacted with a request for these data, but none replied.

Point estimates for each parameter were calculated using a standard confusion matrix where necessary. Confidence intervals (CI) were generated by bootstrapping with the *Boot R-package*. Further details regarding R-scripts and data tables can be found in the Supplementary Information.

## Results

### Search results

The final search yielded a total of 2019 references. All references were imported to EndNote 20, and a total of 525 duplicates were removed, leaving 1494 unique references. The titles of these references were surveyed, along with the abstracts of those assumed to be relevant based on the title. We identified 21 references that fitted the inclusion criteria in Table 1, and for 20 of these we were able to obtain the full text. See Fig. 2 for a general overview of the search results and our selection process, and Table 1 for details regarding the inclusion and exclusion criteria. The results are summarized in Table 3, and the critical appraisal of these studies can be found in Table 2.

**Table 3:** Summary of study methods and results.

Study	Tumor type, stage, and other characteristics	Treatments tested	Brief description of methods	PDO response evaluation	Patient response evaluation	AUC of ROC	Acc / sens / spes / PPV / NPV as supplied by authors	Main conclusions	Other observations
Vlachiogannis et al.	Stage IV colorectal and gastroesophageal cancer from patients enrolled in clinical trials	Library of 55 drugs including classic chemotherapies and targeted drugs	Tumor samples were collected by image-guided biopsies, and cultivated according to organoid protocols. Drug exposure lasted 6–8 days, depending on growth rate	CellTiter Blue cell viability assay, and calculation of relative viability to vehicle control	RECIST criteria	n.a	n.a. / 100% / 93% / 88% / 100%	Organoids derived from patients deemed sensitive to a drug have a much lower GI50-concentration than organoids derived from unsensitive patients	The organoids recapitulated several known genotypes and drug interactions, but mutational profiles could not explain all observed organoid and patient responses
Ganesh et al.	Rectal cancer treatment naïve and pretreated patients, with and without metastases	FOLFOX, cetuximab, and radiation therapy	Tumor samples and adjacent rectal mucosa was collected either by endoscopic or surgical biopsy, and then cultivated first according to organoid protocols, and upon expansion cultivated in a simplified growth medium. Drug exposure lasted 6 days, and samples were exposed to a single dose of radiation	CellTiter-Glo cell viability assay, and calculation of AUC for the dose–response curves of the tested drugs	PFS for chemotherapy response, tumor circumference change upon endoscopy for radiation therapy response	n.a	n.a	For chemotherapy, a *Spearman’s rank correlation coefficient* of 0.86 was observed for PFS and drug response computed as the AUC over the dose–response range, where low AUC correlated with longer PFS For radiation, high organoid sensitivity to radiation correlated with decreased circumference on endoscopy	Organoid response to EGFR-inhibition corresponds with KRAS mutational status
Ooft et al.	Stage IV colorectal cancer from previously untreated patients	Irinotecan, FOLFOX, and FOLFIRI	Tumor biopsies were collected before start of treatment, and cultivated according to organoid protocols. Organoids were exposed to drugs for 6 days	CellTiter-Glo cell viability assay, and calculation of GR50^33^ (dose required to reduce growth rate by 50%) of the tested drugs	RECIST criteria applied to biopsied lesions	0.96 for Irinotecan classifier, and 0.89 for the FOLFIRI classifier	n.a	GR-scores differed significantly in tumoroids derived from PD vs. PR/SD patients. For irinotecan a GR-score > 0.76 classified resistance, while for FOLFIRI a GR-score > 0.46 classified resistance Both classifiers correctly identified all responders, and only misclassified one non-responder for each treatment	The organoid assay had no predictive value for FOLFOX treatment
Pasch et al.	Several different cancer types and histologies	5-FU and oxaliplatin	Tumor biopsies were collected by needle or surgical biopsies and cultivated according to organoid protocols. Drug exposure lasted two days, a period in which the organoids were also subjected to radiation therapy	Change in organoid diameter upon microscopy and calculation of *optical redox ratio*, referring to microscopic NADPH and FAD fluorescence upon excitation, using Glass delta^34^ to calculate effect size	RECIST criteria and CEA-levels of patients	n.a	n.a	PDOs accurately model the tumor they were derived from histologically and genetically, and show heterogenous responses to chemoradiation Based on the organoid drug screen, a patient with metastatic CRC was successfully treated with FOLFOX, achieving SD for more than a year	Organoid viability can be measured by non-invasive methods
Yao et al.	Locally advanced rectal cancer, stage II-III, treatment naïve, mostly adenocarcinoma	5-fluoruracil, irinotecan, and radiation therapy (NACR)	Tumor samples were collected by endoscopic biopsy, and cultivated according to organoid protocols. Drug exposure lasted 6 days, and radiation therapy was given as a single dose. Response evaluation continued for 24 days after exposure	Calculation of organoid size ratio (size at day 24/size at day 0), as well as measuring viability using CellTiter-Glo 3D cell viability assay. Organoids were imaged and assessed for viability every three days	AJCC tumor regression grade as well as clinical response and progression free survival, where a good response is defined as TRG 0 or 1, or complete clinical response	n.a	84.43% / 78.01% / 91.97% / n.a. / n.a. for prediction of patient clinical response	The organoids accurately model patients’ responses to chemoradiation	Organoid viability can be measured by non-invasive methods
Janakiraman et al.	Locally advanced rectal cancer, stage 2 and 3, previously untreated	5-fluoruracil and/or cetuximab, and radiation therapy	Biopsies were obtained during the endoscopic staging procedure, and cultivated according to organoid protocols. Patient derived xenografts were established alongside PDOs. Drug exposure lasted 7 days, and the tumoroids were exposed to daily doses of radiation for the first 5 days	CellTiter Blue cell viability assay, and relative viability compared to vehicle control	AJCC tumor regression grade	n.a	n.a	Treated tumoroids derived from patients achieving TRG0 or TRG1 were significantly less viable after treatment than untreated controls, which was not the case for tumoroids derived from patients achieving TRG2, with one exception	Organoid response to cetuximab corresponded with organoid KRAS status PDX can be utilized to expand organoids upon a limited amount of patient material
Narashiman et al.^19^	Stage IV CRC from patients with peritoneal metastasis	Library of drugs including classic chemotherapies and targeted drugs	Samples were obtained during surgery or as cutaneous biopsies, and cultivated according to organoid protocols. Drug exposure lasted for 6-days before viability was assessed	Cell-Titer-Glo 2.0 cell viability assay, and calculation of AUC of the dose–response curves for the tested drugs	Not specified, but assumed to refer to the RECIST criteria due to the use of “stable disease”	n.a	n.a	Organoid sensitivity could not predict sensitivity to FOLFOX Two patients were treated with targeted drugs according to the organoid screen, where one patient achieved 3 months of stable disease before progressing, and one patient died four weeks after treatment with no sign of response	Organoid sensitivity to drugs was consistent with genomically predicted targets, but could not explain all responses/non-responses
Xu et al.	Metastatic colorectal cancer, stage IV	FOLFOX and FOLFIRI	Samples were obtained by biopsy of liver metastases, and cultivated according to organoid protocols with the use of a cellulosic sponge as extracellular matrix. Drug exposure lasted 6 days	CellTiter-Glo 3D cell viability assay, and calculation of IC50 and AUC of the dose–response curves for the tested drugs	RECIST	n.a	n.a	PDOs derived from responsive patient and exposed to FOLFOX, grown in the cellulosic sponge model, had significantly lower AUC under the DRC and IC50 compared to PDOs derived from non-responsive patients. For PDOs cultivated in Matrigel, there was no statistically significant difference in viability between PDOs derived from responsive and non-responsive patients For FOLFIRI treatment, both conditions modelled patient responses	PDOs cultivated in Matrigel showed signs of epithelial-mesenchymal transition, which was not the case in the cellulosic sponge model
Wang et al.	Metastatic colorectal cancer, stage IV	FOLFOX, FOLFIRI, and FOLFOXIRI	Samples were collected by surgical- or core-needle biopsy and cultivated according to organoid protocols (medium composition is not disclosed). Drug exposure lasted 4 days	CellTiter-Glo 3D cell viability assay, and calculation of IC50 of the tested drugs	RECIST criteria and AJCC tumor regression grade	0.884 for the IC50 classifier	79.69% / 63.33% / 94.12% / 90.48% / 74.42%	The organoid IC50 classifier for treatment sensitivity generated in the pilot-study accurately classified patients in the blinded study	n.a
Ooft et al.^21^	Metastatic colorectal cancer, stage IV	vistusertib, capivasertib, selumetinib, gefitinib, palbociclib, axitinib, gedatolisib, and glasdegib; drugs approved by the FDA, or in late clinical trials	Tumor samples were obtained with core-needle biopsies and cultivated according to organoid protocols using a simplified medium composition. Drug exposure lasted 6 days before viability was assessed. Where a match existed, eligible patients were treated with the drug in monotherapy	CellTiter-Glo 3D cell viability assay, and calculation of GR50 of the tested drugs	RECIST criteria	n.a	n.a	The matched treatment was not effective in any of the 6 eligible patients, and the trial was discontinued	n.a
Park et al.^22^	Rectal adenocarcinoma, stage III to IV	Radiation therapy	Samples were obtained by endoscopic biopsy and cultivated according to organoid protocols. Days of exposure to radiation is not disclosed, but viability was assessed 7 days after start of treatment, and survival fraction 14 days after start of treatment. Machine learning was applied using the survival fraction data to build predictive models for TRG outcomes	Surviving cell fraction counted by bright field microscopy (# of formed organoids/# of plated cells) and viability as assessed by the CellTiter 96 AQUEOUS One Solution	AJCC tumor regression grade	Out of several iterations, the best achieved was 0.918 for good responders (TRG 0) and 0.971 for poor responders (TRG 3)	81.5%/n.a./n.a./n.a./n.a. for good responders and 92.1%/n.a./n.a./n.a./n.a. for poor responders	The surviving cell fraction was found to be the best measure to differentiate between sensitive and resistant samples, and the model based on these data accurately classified patients as good or poor responders	n.a
Cui et al.^11^	Colorectal cancer, stage III-IV, adenocarcinoma	5-FU, oxaliplatin, Xeliri, FOLFOX, FOLFIRI, FOLFOXIRI, cetuximab, fruquintinib, and regorafenib	The method for obtaining tumor tissue is not described. PDOs were cultivated according to organoid protocols, with the use of a nested array chip plate instead of a conventional microwell plate. Drug exposure lasted 5 days	CellTiter-Glo 3D cell viability assay, and calculation of the IC50 of the tested drugs	RECIST criteria	n.a	n.a	The three included patients received FOLFOX treatment, and experienced tumor shrinkage. Organoids derived from these patients were sensitive to the same regimen, where FOLFOX (among others) showed the lowest IC50 of the tested drugs and regimens	Using a nested array chip sped up organoid growth, reduced Matrigel consumption, and reduced variation in size and cellular activity of the tumoroids compared to conventional microwell plates
Cho et al.^10^	Colorectal cancer, stage I-IV, mainly untreated	5-FU, oxaliplatin, SN-38, and cetuximab	Both tumor and normal tissue samples were obtained by surgical biopsies, and cultivated according to organoid protocols. Drug exposure lasted 6 days	CellTiter-Glo 3D cell viability assay, and calculation of the IC50 of tested drugs, as well as AUC of the dose–response curves Additionally, an “organoid score” was defined, where a higher AUC of the DRC gives a higher score	RECIST-criteria and progression free survival	n.a	n.a	Organoids derived from patients responding to treatment were more sensitive to the treatment in question than organoids derived from insensitive patients An organoid score of 2.5 was found to best differentiate between sensitive and resistant organoids, and the organoid score correlated with progression free survival	DNA analysis revealed RNF43 gene mutations in several organoids, which were highly sensitive to porcupine O-acyltransferase (PORCN) inhibitor treatment
Ding et al.^12^	Colorectal cancer, adenocarcinoma, stage IV	Oxaliplatin	Droplet emulsion microfluidics to generate micro-organospheres (MOSs) from low-volume patient tissues, which was subsequently subjected to oxaliplatin and compared to patient responses retrospectively	CellTiter Glo, and calculation of percent cytotoxicity following the formula of: 100*(1-(average CellTiterGlo^drug^/ average CellTiterGlo^control^))	Tumor size change on CT-scan, assumed to refer to the RECIST criteria	n.a	n.a	MOS could predict 3/4 clinically responding patients, and 3/4 non-responding patients withing a timeframe of less than 14 days	MOS are more suitable for assessing the potency of adoptive cellular therapies using T-cells (immunotherapy) than more traditional ways of cultivating organoids
Hsu et al.^14^	Rectal cancer, samples obtained prior to neoadjuvant chemoradiation	Radiotherapy	PDOs were generated following organoid protocols, and organoids were exposed to radiation using a single hit multi-target algorithm to generate a dose (D_0_) required to reduce survival fraction to 37%. PDO and patient response was compared prospectively	Manual counting of surviving organoids, calculation of survival fraction compared to vehicle control, and generation of dose–response curves based on the data	RECIST criteria and tumor circumference change upon surgery	n.a	n.a	A lower D_0_ was associated with both better clinical outcome for the patients, as well as a larger reduction in tumor size	Olaparib radiosensitized the PDOs of two patients who experienced local recurrence after neoadjuvant chemoradiation
Mo et al.^18^	Stage IV CRC with liver metastases, mostly moderately differentiated adenocarcinoma	5-FU, oxaliplatin, and irinotecan	PDOs were generated from matching CRC and liver-metastasis tissue according to organoid protocols, and subjected to the same chemotherapy regimen as the patient they were derived from (FOLFOX/FOLFIRI)	CellTiter-Glo 3D cell viability assay, and IC50 of the tested drugs	RECIST criteria	0.850/0.714 and 0.920/0.750 for FOLFOX and FOLFIRI, respectively, with the first number representing clinical response, and the second progression free survival	n.a	IC50 of organoids derived from patients with stable disease/partial response was significantly different from those derived from patients with progressive disease	n.a
Lv et al.^16^	Locally advanced rectal adenocarcinoma, TNM stage I-IV	5-FU, irinotecan, and radiation	Tumor cells were isolated upon diagnosis and cultivated according to organoid protocols before treatment was given. Patients were divided into a training-cohort and a validation-cohort	Calculation of organoid size ratio (size at day 24/size at day 0), as well as measuring viability using CellTiter-Glo 3D cell viability assay. Organoids were imaged and assessed for viability every 3 days	Clinical complete response evaluated by digital rectal examination, endoscopy, and MRI, as well as AJCC TRG classification	The OSR had an AUC for predicting complete response of 0.8276 (95% CI 0.7234 − 0.9319) in the training cohort and 0.7963 (95% CI:0.5974 − 0.9952)	n.a	Organoid and patient response matched in 73.8% of cases for irinotecan, 71.3% of cases for irradiation, and 66.3% for 5-FU. Patients with organoids sensitive to irinotecan and radiation had more complete clinical response, and longer disease free- and metastasis free survival. Patients with organoids sensitive to 5-FU had more complete clinical response, but no differences in disease free- and metastasis free survival	n.a
Tang et al.^24^	Stage II-IV colorectal cancer, both mucinous- and adenocarcinoma	5-FU or capecitabine in combination with oxaliplatin (FOLFOX/XELOX), in addition to other drugs not used in response prediction	Tumor samples were harvested by surgery or biopsy, and cultured according to organoid protocols, and exposed to drugs for 96 h before assessing viability. Patients were treated according to MDT decisions	CellTiter-Glo 3D cell viability assay, and IC50 of the tested drugs	Size-change of lesions upon image diagnostics (assumed to refer to the RECIST criteria), AJCC/CAP tumor regression grade, and CEA-levels. CR/PR, AJCC/CAP 0–1, or decreasing CEA-levels were defined as clinical response	0.771 for the IC50 of the organoids, and 0.798 for the AUC of the dose–response curves of the organoids	75%/75.36%/74.68%/72.22%/77.63%	Organoid and patient response was largely similar, and the optimal IC50 value to distinguish sensitive patients from resistant was 43.26 μmol/L for the FOLFOX regimen, drug not specified	Progression free survival in metastatic CRC patients was significantly worse in patients with organoids insensitive to treatment than in those sensitive to treatment, as indicated by the IC50 cutoff value (median 8 vs. 11 months)
Martini et al.^17^	Stage IV colorectal cancer, not further specified	5-FU, oxaliplatin, irinotecan, cetuximab, encorafenib, trastuzumab, lapatinib, regorafenib for the first cohort, with an added number of targeted therapies in cohort 2	Tumor samples were harvested by core-needle biopsies and cultured according to organoid protocols. Organoids were cultivated with drugs for 6 days before readout. The second cohort was supplemented with genotyping of 431 pan-cancer genes	For the first cohort an MTS-assay was applied. For the second cohort the CellTiter-Glo cell viability assay was used. For both, relative viability compared to vehicle control was calculated	Is not specified, but due to the use of “stable disease” it is assumed to refer to the RECIST criteria	n.a	n.a	In cohort 1 they found a heterogeneous response rate to the examined drugs, as would be expected in clinical practice. They found a “direct correlation of drug sensitivity between patients’ treatment and organoid treatment”, without specifying further In cohort 2, they used genotyping and the organoid assay to assign treatment to two patients, achieving stable disease for 5 and 6 months	n.a
Wang et al.^27^	Stage IV colorectal cancer, both adenocarcinoma and mucinous carcinoma	FOLFOX and FOLFIRI	Tumor samples were obtained from patients undergoing surgery and cultivated according to organoid protocols, and later exposed to drugs. Patients were treated according to clinical guidelines	CellTiter-Glo 3D cell viability assay, and IC50 of the tested drugs	RECIST criteria, as well as measrement of CEA levels	The drug response alone had an AUC of 0.731 for predicting 1-year progression free survival. When combined with tumor location, histological subtype, and R0-resection status, the AUC increased to 0.901	n.a	The drug sensitivity of a patient’s organoids, in combination with other patient-specific factors, can predict the probability of a patient experiencing 1-year progression free survival or not	n.a

*“Organoid protocols”* refer to the key method steps outlined in Fig. 1.

*Acc* accuracy, *sens* sensitivity, *spes* specificity, *PPV* positive predictive value, *NPV* negative predictive value, *AUC of ROC* area under the curve of the receiver operating characteristic curve, *IC50* dose of drug required to reduce viability of sample to 50% of control, *GR50* dose of drug required to reduce growth rate of sample to 50% of control.

### Organoid cultivation methods

All included references employed similar and comparable cultivation methods, with smaller variations in non-critical steps. The general procedure follows a series of steps: (1) collection of cancer material through biopsy or surgical removal, (2) mechanical and/or enzymatic digestion of the cancer tissue, (3) embedding of the cancer tissue in a suitable supporting growth environment (e.g. Matrigel or other forms of extracellular matrices), (4) addition of more or less chemically defined growth medium to allow formation and growth of organoids, (5) exposure to drugs, and (6) assessing the response. Details regarding methodologies can be found in Supplementary Table S5.

Common areas of variation were seeding densities of cells/tumour fragments, medium composition, type of extracellular matrix, drugs used, duration of drug exposure, and method of organoid response readout.

The study by Janakiraman et al.^15^ deviated somewhat from the most common approach to organoid formation, as they used the tumour samples to first establish patient derived xenografts (PDX) in mice. They then used tumour material harvested from the xenografts to cultivate organoids. The authors present this as a way to expand available material from a sparse initial amount, which can be a limiting factor when dealing with core needle biopsies. Other studies used self-fabricated plates to make automation easier^11^, generated *micro organospheres* by pumping a Matrigel-cell suspension into oil^12^, or used alternatives to basement membrane extracts (Matrigel, Geltrex, etc.) for cultivation^28^.

### Use of clinically approved therapies

All studies identified by the literature search tested therapies approved for treatment of colon and/or rectal cancer. Patients were treated according to clinical guidelines, or with off-label or investigative agents, sometimes being included in other clinical trials to get access to drugs. Organoids were treated with both single drugs and clinically approved combinations, as well as radiation therapy. The most frequently tested therapies were 5-FU alone, and its combinations with either oxaliplatin or irinotecan (or its active metabolite SN-38), which comprise the most commonly used standard-of-care chemotherapy regimens in the treatment of CRC. Additionally, several of the included references^10,11,15,17,19,21,25^ tested targeted therapies (monoclonal antibodies, tyrosine-kinase inhibitors), all of which were either clinically approved for the treatment of CRC or other cancers, or investigative therapies in clinical trials.

### Methods of response evaluation

The included studies used different means of comparing patient and organoid response. Patients were evaluated according to established clinical criteria (Response Evaluation Criteria in Solid Tumours, abbreviated RECIST^35^, and American Joint Committee on Cancer Tumour Regression Grade, abbreviated AJCC-TRG^36^), progression free survival, and/or tumour circumference change on endoscopy. Organoid response was evaluated most commonly by the *CellTiter Glo viability assay*, while two of the included studies also used the *CellTiter Blue viability assay*^37^. Most studies reported either relative viability compared to vehicle control, or IC50 of the tested drugs.

Interestingly, five of the included studies^14,16,22,23,29^ applied non-invasive, continuous readouts of organoid viability. These studies used brightfield microscopy images to assess changes in organoid diameter, and calculated the percentage change in growth from seeding, based on the assumption that organoid growth correlates with treatment response. The Pasch et al.^23^ study additionally utilised *optical metabolic imaging* to capture NAPDH and FAD fluorescence, which was used to calculate a *redox ratio.* This provides a measure of cellular metabolic activity without using dyes or disrupting the organoids^38^, and has been shown to accurately predict drug response in patient derived cellular models^39^.

### Correlation between patient and organoid response

Despite differences in the evaluation of organoid and patient response to therapy, all of the included studies reported that organoid response to treatment was mainly similar to patient response to treatment, with the exception of the 2021 study by Ooft et al.^21^, where responses to targeted therapies were found to correlate poorly between organoids and patients.

Briefly, Janakiraman et al.^15^ found that for organoids derived from patients with tumour regression grade (TRG) 0 or 1 (complete and partial response, respectively, as determined by histology assessments) there was a significant difference in viability between drug-treated and control organoids. This was not the case for patients with TRG 2 (minimal response), indicating that organoids from patients with low response to clinically administered therapies also were relatively insensitive to the drug in the paraclinical setup. Ganesh et al.^13^ found that the area under the curve (AUC) of organoid dose–response curves were inversely correlated with progression free survival of patients, and directly correlated with the change in tumour circumference on endoscopy. Xu et al.^28^ observed a notable difference in both the AUC and the concentration of drug required to decrease viability of organoids to 50% of control (IC50) in their cellulosic sponge model. This discrepancy was observed when comparing organoids derived from patients with partial response or stable disease to those from patients with progressive disease. Vlachiogannis et al.^25^ reported that in total, their screens show 100% sensitivity, 93% specificity, 88% positive predictive value, and 100% negative predictive value. It is however not explicitly stated how organoids were classified as drug sensitive or resistant. Narashiman et al.^19^ found that the drug vandetanib dramatically reduced the viability of organoids from one patient, and this patient was subsequently assigned to the drug. The patient unfortunately showed no signs of response and died four weeks after initiation of treatment. The organoids of another patient showed sensitivity to gemcitabine, and was assigned to this treatment, achieving stable disease for three months. Cui et al.^11^ followed three patients who experienced tumour shrinkage on FOLFOX treatment, and found that their organoids were sensitive to the same treatment. Hsu et al.^14^ found that for organoids where a lower dose of radiotherapy was required to reduce the surviving cell fraction to 37%^40^, the patients from whom they were derived tended to experience larger tumour shrinkage in a clinical setting. Mo et al.^18^ found that the IC50 of organoids derived from patients with stable disease or partial response to treatment was significantly lower than that of organoids derived from patients with progressive disease. Martini et al.^17^ used the viability data from their organoid assays to assign two patients to treatment. The first patient received FOLFOX + bevacuzimab and achieved stable disease for 6 months, while the other patient received mitomycine + capecitabine, achieving stable disease for 5 months. These observations are summarized in Table 3.

The remaining studies all devised a classifier for organoid sensitivity, i.e. a threshold below which an organoid is deemed sensitive to the given treatment, by measuring organoid growth or viability and comparing these data to patient responses. Pasch et al.^23^ utilised their classifiers for both diameter change and redox ratio to successfully predict one patient’s response to the FOLFOX chemotherapy regimen prospectively. Ding et al.^12^ found that organoid response matched patient responses in six out of eight cases. The 2019 study by Ooft et al.^20^, Yao et al.^29^, both the 2021 and 2023 study by Wang et al.^26,27^, Park et al.^22^, Cho et al.^10^, Lv et al.^16^, and Tang et al.^24^ applied their classifier to larger cohorts of patients. Ooft et al. and Wang et al. both used an IC50 cut-off value, while Yao et al. used an organoid diameter change cut-off. Yao et al. and Ooft et al. compared patient and organoid responses to find a mean optimal cut-off, which best separated responsive PDOs and responsive patients from resistant PDOs and non-responsive patients. In contrast, Wang et al. first conducted a pilot study with a smaller cohort of patients to establish their classifier threshold, which they then validated in a larger cohort in a blinded study. All three studies concluded that their classifiers performed well. Park et al. found that the surviving cell fraction was best able to differentiate between sensitive and resistant samples, and that it accurately classified responders- and non-responders. Cho et al. found that an organoid score of 2.5 (see Table 3 for definition) accurately differentiated between sensitive and resistant organoids, and that a lower score correlated with longer progression free survival. Lv et al. found that there was a match rate between patient and organoid response of 73.8%, 71.3%, and 66.3% for irinotecan, radiation, and 5-FU respectively. Tang et al. found that a lower IC50 for the FOLFOX regimen was associated with better patient outcomes. Finally, Wang et al. found that the drug sensitivity of organoids could predict the probability of a patient experiencing a 1-year progression free survival or not.

### Meta-analysis of organoid assays

**Figure 3:**
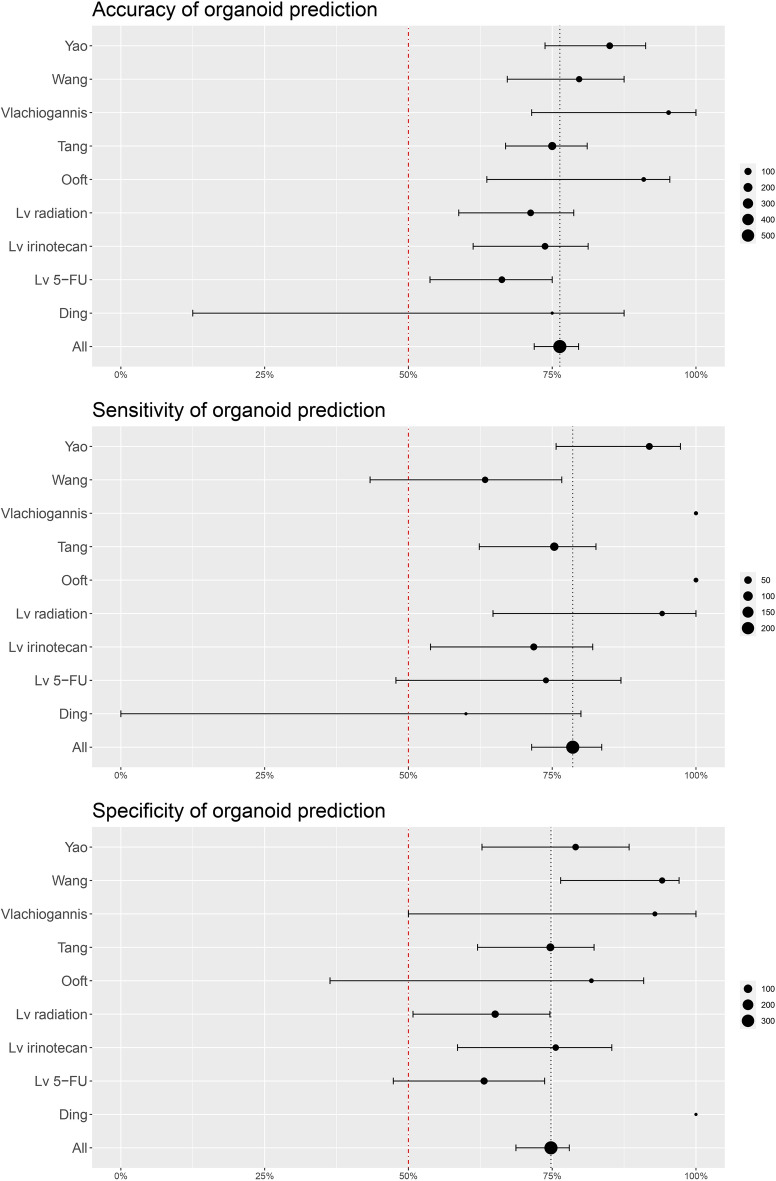
Accuracy, sensitivity, and specificity of the organoid assays. Point estimates and 95% confidence intervals are shown. Size of the dots corresponds to the number of patients. The vertical red lines represent 50%, which would be equivalent to a random draw.

**Figure 4:**
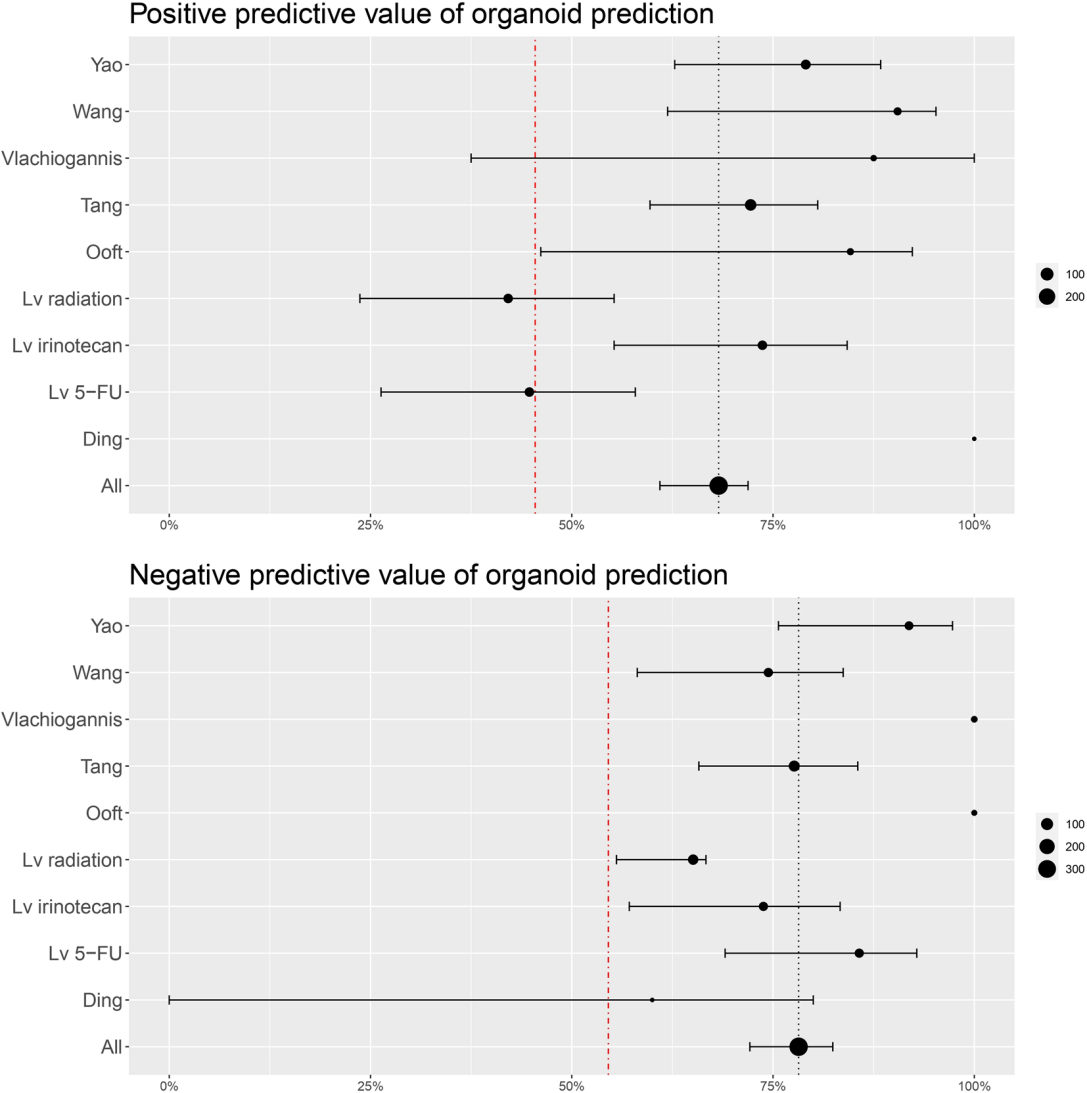
Positive and negative predictive values for the organoid assays. Point estimates and 95% confidence intervals are shown. Size of the dots correspond to the number of patients. Vertical red lines represent the pre-test likelihood of a patient responding to treatment (for PPV) and not responsding to treatment (for NPV).

Several of the included studies^12,16,20,24–26,29^ provided measures of (or the numbers needed to calculate) accuracy, sensitivity, specificity, positive predictive value (PPV), and negative predictive value (NPV) of the organoid assays, when compared to patient responses. These studies were included in our meta-analysis to find predictive performances for individual studies and for all studies combined, as shown in Figs. 3 and 4. Overall, the organoid assays of the included studies show high predictive performance in identifying patients that respond to a given therapy, with some variation. Bootstrapping was applied to calculate confidence intervals. Point estimates with confidence intervals for individual studies are summarised in Supplementary Table S6. We found an overall test accuracy of 0.76 (95% CI 0.72–0.80), a sensitivity of 0.79 (95% CI 0.71–0.84), and a specificity of 0.75 (95% CI 0.69–0.78). Combined, the assays had both positive and negative predictive values exceeding the pre-test likelihood of response to treatment of 47%, with gathered means of 0.68 (95% CI 0.61–0.72) for PPV and 0.78 (95% CI 0.72–0.82) for NPV.

## Discussion

In this review we present an overview of the growing field of translational organoid research, with the main finding being that organoids hold great potential in predicting patient responses to radio- and chemotherapy in CRC.

Although the use of organoids in translational medicine is still very much in its early stages, several reviews have been published on the subject, including some that align with the focus of the present review^31,41,42^. Our systematic review provides a more comprehensive meta-analysis compared to previous reviews, where we have also included the computation of PPV and NPV.

It is a growing concern for many that there are no standardised methods for the cultivation of organoids, a fact that has led to a lack of reproducibility of data originating for experiments with 3D models, as well as heterogeneity in both organoid morphology and response to treatments^43,44^. Although our included references mainly retained the same key steps in their cultivation protocols, they too differ in the details. Despite these differences, all the references included in our paper report a positive correlation between organoid and patient response to the same treatment, with the exception of the 2021 study by Ooft et al.^21^. There is however considerable variability of these results, even for studies that use near identical protocols, such as Lv et al.^16^ and Yao et al.^29^. Although it is likely that standardisation of cultivation protocols will lead to more consistent data across different labs, the methods of today are still able to capture the predictive ability of organoid models. We take the fact that research groups using near identical cultivation protocols report different results to imply that there are likely other factors than differences in cultivation protocols that also contribute to the observed heterogeneity in the predictive power of organoid assays.

Most of the identified references focus on standard-of-care chemotherapies (5-FU, oxaliplatin, irinotecan, radiation). This is also true for the studies included in our meta-analysis, with the exception of the Vlachiogannis et al.^25^ study, which utilised a broad panel of both chemotherapies and targeted inhibitors. The use of standard-of-care and clinically approved therapies likely makes the road to clinical trials and possible clinical implementation of organoid-based diagnostics shorter than for therapies which are not routinely used in the clinic.

Several of the papers also delved into genotype–phenotype investigations, exploring how known genetic alterations affect PDO response to targeted therapies for specific alterations. Vlachiogannis et al.^25^ showed how specific amplifications and activating mutations in their organoids correlated with higher sensitivity to therapies specifically targeting these alterations. Interestingly, they found that this was not true for all mutations included in their next generation sequencing panel, which could highlight the importance of the PDOs as a preclinical model that circumvents the intricacies of using genomic data to match patients with effective therapies^5^. Additionally, the studies by Ganesh et al.^13^ and Janakiraman et al.^15^ specifically compared organoid *KRAS* mutation status to cetuximab response. Both found that *KRAS*-mutation conferred a lack of response to treatment with the EGFR-inhibiting antibody, as would be expected from clinical observations.

Interestingly, Ooft et al.^20^ found that their PDOs could predict sensitivity to irinotecan and FOLFIRI but not FOLFOX. The study by Xu et al.^28^ directly addressed this observation. They suggest that Ooft et al. were unable to predict the activity of FOLFOX due to *Matrigel* inducing the epithelial-to-mesenchymal transition (EMT) in the organoids, as evident by reduced expression of E-cadherin on Matrigel-cultivated PDOs. Reduced E-cadherin expression is a commonly recognized marker of EMT^45^. They find that by using their cellulosic sponge model, PDOs maintain expression of E-cadherin, while simultaneously accurately modelling patient response to FOLFOX. However, several of the other included studies cultivated their PDOs in Matrigel, without taking measures described by Xu et al. and still found a correlation between patient and PDO response to FOLFOX, which indicates that also other factors could contribute to the lack of correlation found by Ooft et al. Their medium composition, for example, is different from most other included studies, shared only with Tang et al., which had one of the lowest predictive performances of our included studies. These discrepancies call for further explorations into medium composition and its influences on drug responses.

Our meta-analysis (Fig. 4) shows a pooled PPV of 68% (95% CI 0.61–0.72) and NPV of 78% (95% CI 0.72–0.82). The combined response rate of patients in our included studies was 47%, which matches clinically observed response rates in meta-analyses of standard colon cancer therapy^46^. This means that using organoids to guide treatment decisions could significantly increase response rates.

A common drawback for several of the included studies is their *retrospective* nature, i.e., that organoid drug screens were not used to assign patients to specific treatment regimens, but rather PDO and patient sensitivity to treatment are compared after therapy is given. As such, retrospective studies of organoid responses could be said to model, rather than predict, treatment responses. Four of the included studies did however make use of the PDO assays to assign patients to treatment^17,19,21,23^, and for the majority of these successfully so. Other studies included follow-up data for their patients, showing how organoid responses correlated positively with the progression free survival of included patients^10,16,24,27^, further strengthening the predictive potential of PDOs.

Several of the included studies point to larger prospective studies in the vein of Pasch et al.^23^ as a natural next step. Ooft et al.^21^, following up on their work from 2019, used PDO screens to prospectively assign 6 patients to single agent off label *targeted* therapies. Unfortunately, the patients did not respond to the assigned treatments, despite their organoids being classified as sensitive, for the particular drugs tested. However, it is important to note that monotherapy with targeted agents in CRC has previously been shown to be suboptimal, as the effect can be circumvented by upregulation of alternate cellular signalling pathways^47^. This should serve as a reminder that while organoids are a huge step forward compared to simpler cellular models, they still cannot recapitulate the entire complexity of the cancer disease.

## Concluding remarks

To answer our initial question, on whether cancer organoids can predict treatment outcome for anti-cancer therapies in CRC, we conclude that our meta-analysis demonstrate a large potential for the use of PDOs to assist treatment decisions. The included studies have focused mostly on standard-of-care chemotherapy regimens, along with radiation therapy, which is an area with a severely unmet clinical need in patient treatment response prediction. Although the influence of a publication bias cannot be excluded, seeing as near all the included studies report positive correlations, it does indeed seem like cancer organoids are well suited to predict treatment outcomes for anti-cancer therapies in CRC. Larger and prospective studies are needed, as well as establishing standardised cultivation protocols, organoid viability assays, and one or several validated classifiers for organoid sensitivity that accurately predicts treatment response. Several of the included studies point to prospective studies using PDOs to assign patients to standard-of-care systemic therapies as their next step, something that would be of great benefit for patients with metastatic colorectal cancer, relying on systemic treatment for survival.

### Supplementary Information

Supplementary Information 1.

Supplementary Information 2.

Supplementary Information 3.

Supplementary Information 4.

## Data Availability

All data generated or analysed during this study are included in this published article and its supplementary information files. For further clarification, information, or other remaining questions regarding the data and analysis thereof, the corresponding author can be contacted.
