# Supplementary material for: Systematic review: predictive value of organoids in colorectal cancer
Source: Sci Rep. 2023 Oct 23;13:18124. doi: 10.1038/s41598-023-45297-8 (PMC10593775; doi:10.1038/s41598-023-45297-8)
Supplement: Supplementary file 4 — Supplementary Information 4. [file 41598_2023_45297_MOESM4_ESM.pdf]

Systematic review: Predictive value of organoids in colorectal cancer

B. Cristoffer Sakshaug[1], Evelina Folkesson[1], Tonje H. Haukaas[2], Torkild Visnes[2], Åsmund Flobak[1,3]

1. Department of Clinical and Molecular Medicine, Norwegian University of Science and Technology, Trondheim, Norway
2. Department of Biotechnology and Nanomedicine, SINTEF Industry, Trondheim, Norway
3. The Cancer Clinic, St Olav's University Hospital, Trondheim, Norway

Keywords: colorectal cancer, organoids, anti-cancer therapy, treatment response prediction

Corresponding author:
Åsmund Flobak
Address: Prinsesse Kristinas gate 1, 7030 Trondheim, Norway
E-mail: asmund.flobak@ntnu.no,
Phone: +47 72 57 37 17

Supplementary materials

Concept table

From the research question, we drew four main concepts. Both the question, concepts, and search terms are detailed in Supplementary table 1. The first concept was based on organoid/spheroid technology. This refers to a three-dimensional cellular culture, made up of either cell-lines or patient-derived material, where the latter was of interest for this paper. This concept served as the constraint of the search. The second concept encompassed terms to make a connection between the first and third concept, the last-mentioned of which introduced a clinical aspect to the search. The fourth concept involved several different forms of anti-cancer therapies, and synonyms for these. Since radiotherapy is never used alone in the treatment of CRC, it was only included when in combination with chemotherapy.

The terms within each concept were combined with the "OR" Boolean operator, and the concepts themselves were then combined with the "AND" Boolean operator.

Supplementary Table S1, research question and concept table for literature search.

Research question: "*Can cancer organoids predict treatment outcome for anticancer therapies?* "

**Organoids**	**Predict**	**Treatment outcome**	**Anticancer therapies**
Organoid(s) (Em/Med)	Translational medical research (Med)	Treatment outcome (Med/Em)	Antineoplastic agents/agent (Med/Em)
Spheroids, cellular (Med)	Translational research (Em)	Treatment response (Em)	Radiochemotherapy (Em)
Tumor spheroid (Em)	"Translational medical research" (ft)	"Treatment outcome*" (ft)	Chemoradiotherapy (Med/Em)
Multicellular spheroid (Em)	"Translational research" (ft)	"Treatment response*" (ft)	Chemotherapy (Em)
"Organoid*" (ft)	"Predict*" (ft)	"Patient response*" (ft)	Cytostatic agents (Med/Em)
"Spheroid*" (ft)	"Mimic*" (ft)		Molecular targeted therapy (Med/Em)
	"Model*" (ft)		"Antineoplastic agent*" (ft)

			“Chemoradiotherap*” (ft)
			“Radiochemotherap*” (ft)
			“Cytostatic agent*” (ft)
			“Chemotherap*” (ft)
			“Molecular targeted therap*” (ft)
Med = MeSH terms from MEDLINE, Em = Emtree terms from Embase, ft = free text/keyword			

Pubmed search

The Pubmed search strategy is shown in *Table 5.* The terms used for this search consisted of the MeSH thesaurus terms (Med), and all free text terms listed. No limitations were placed on the search. In addition to this, one paper was obtained by hand-search.

**Supplementary Table S2,** search terms for Pubmed search.	
**Search #**	**Query**
1	“Organoids”[MESH]
2	“Spheroids, cellular” [MESH]
3	“Organoid*”
4	“Spheroid*”
5	“Translational Medical Research”[MESH]
6	“Translational Medical Research”
7	“Translational research”
8	“Predict*”
9	“Mimic*”
10	“Model*”
11	“Treatment Outcome”[MESH]
12	“Treatment outcome*”
13	“Treatment response*”
14	“Patient response*”
15	“Antineoplastic Agents”[MESH]
16	“Chemoradiotherapy”[MESH]
17	“Cytostatic Agents”[MESH]
18	“Molecular Targeted Therapy”[MESH]
19	“Antineoplastic agent*”
20	“Chemoradiotherap*”
21	“Radiochemotherap*”
22	“Cytostatic agent*”

23	“Molecular targeted therap*”
24	“Chemotherap*”
25	1 OR 2 OR 3 OR 4
26	5 OR 6 OR 7 OR 8 OR 9 OR 10
27	11 OR 12 OR 13 OR 14
28	15 OR 16 OR 17 OR 18 OR 19 OR 20 OR 21 OR 22 OR 23 OR 24
29	25 AND 26 AND 27 AND 28

Embase search

The Embase search strategy is shown in *Table 6.* The terms used in this search consisted of the Emtree thesaurus terms (Em), and all free text terms listed. No limitations were placed on the search.

**Supplementary Table S3,** search terms for Embase search.	
**Search #**	**Query**
1	organoid*.mp. or exp organoid/
2	exp tumor spheroid/ or Spheroid*.mp.
3	exp multicellular spheroid/ or “Multicellular spheroid*”.mp.
4	“tumor spheroid*”.mp.
5	“translational research”.mp. or exp translational research/
6	predict*.mp.
7	mimic*.mp.
8	model*.mp.
9	“translational medical research”.mp.
10	exp treatment outcome/ or “treatment outcome*”.mp.
11	exp treatment response/ or “treatment response*”.mp.
12	“patient response*”.mp.
13	exp antineoplastic agent/ or “antineoplastic agent*”.mp.
14	exp radiochemotherapy/ or radiochemotherap*.mp.
15	exp chemoradiotherapy/ or chemoradiothrapy*.mp.
16	exp chemotherapy/ or hemotherapy*.mp.
17	exp cytostatic agent/ or “cytostatic agent*”.mp.
18	“molecular targeted therap*”.mp. or exp molecular targeted therapy/
19	1 or 2 or 3 or 4
20	5 or 6 or 7 or 8 or 9

21	10 or 11 or 12
22	13 or 14 or 15 or 16 or 17 or 18
23	19 and 20 and 21 and 22

Scopus search

The SCOPUS search strategy is shown in *Table 7.* The terms included in this search were all free text terms. No limitations were placed on the search.

**Supplementary table S4,** search terms for Scopus search.	
**Search #**	**Query**
1	(TITLE-ABS-KEY (organoid*) OR TITLE-ABS-KEY (spheroid*))
2	(TITLE-ABS-KEY (“translational medical research”) OR TITLE-ABS-KEY (“translational research”) OR TITLE-ABS-KEY (predict*) OR TITLE-ABS-KEY (mimic*) OR TITLE-ABS-KEY (model*))
3	(TITLE-ABS-KEY (“treatment outcome*”) OR TITLE-ABS-KEY (“treatment response*”) OR TITLE-ABS-KEY (“patient response*”))
4	(TITLE-ABS-KEY (“antineoplastic agent*”) OR TITLE-ABS-KEY (hemotherapyrapy*) OR TITLE-ABS-KEY (radiochemotherap*) OR TITLE-ABS-KEY (“cytostatic agent*”) OR TITLE-ABS-KEY (hemotherapy*) OR TITLE-ABS-KEY (“molecular targeted therap*”))
5	((TITLE-ABS-KEY (organoid*) OR TITLE-ABS-KEY (spheroid*))) AND ((TITLE-ABS-KEY (“translational medical research”) OR TITLE-ABS-KEY (“translational research”) OR TITLE-ABS-KEY (predict*) OR TITLE-ABS-KEY (mimic*) OR TITLE-ABS-KEY (model*))) AND ((TITLE-ABS-KEY (“treatment outcome*”) OR TITLE-ABS-KEY (“treatment response*”) OR TITLE-ABS-KEY (“patient response*”))) AND ((TITLE-ABS-KEY (“antineoplastic agent*”) OR TITLE-ABS-KEY (chemotherapyrapy*) OR TITLE-ABS-KEY (radiochemotherap*) OR TITLE-ABS-KEY (“cytostatic agent*”) OR TITLE-ABS-KEY (hemotherapy*) OR TITLE-ABS-KEY (“molecular targeted therap*”)))

Supplementary Table S5. Detailed description of methods, as supplied by authors.

Study	Digestion	Extracellular matrix and plating	Medium composition	Passaging
Vlachiogannis et al. (2018)	Biopsies were minced, conditioned in 5 ml PBS/EDTA 5 mM for 15 min at room temperature, and digested in 5 ml PBS/EDTA 1 mM containing 2x TrypLe for 1 hr at 37°C. Following digestion, mechanical force (pipetting) was applied in order to facilitate cell release in solution.	Growth factor reduced Matrigel.	Advanced DMEM/F12, supplemented with 1x B27 additive, 1x N2 additive, 0.01% BSA, 2 mM L-Glutamine, 100 units/ml penicillin-streptomycin, 50 ng/ml EGF, 100ng/ml Noggin, 500 ng/ml R-spondin 1, 10 nM Gastrin, 10 ng/ml FGF-10, 10 ng/ml FGF-basic, 100 ng/ml Wnt-3A, 1 µM Prostaglandin E_2, 10 µM Y-27632, 4 mM Nicotinamide, 0.5 µM A83-01, 5 µM SB202190.	PDOs were mechanically harvested (pipetting) out of matrigel using PBS-EDTA 1mM containing 1x TrypLe, and incubated for 20 min at 37°C. PDOs were then dissociated to single cells by applying mechanical force (pipetting), washed with HBSS, pelleted (1,200 rpm, 5 min, 4°C).
Ganesh et al. (2019)	Tissue was washed with ice-cold PBS-Abs (phosphate buffered saline with gentamicin and metronidazole) buffer and then chopped into 1-mm pieces in ice-cold PBS-DTT buffer (PBS with 10 mM dithiothreitol). The fragments were digested in a digestion medium consisting of advanced DMEM/F12 with 2% FBS, penicillin-streptomycin, 100 U/ml collagenase type XI, and 125 µg/ml dispase type II	After digestion, biopsied samples were embedded directly in Matrigel, while surgical samples were filtered and pelleted before being embedded in Matrigel.	Advanced DMEM/F12 was supplemented with penicillinstreptomycin, 1 × B27, 1 × N2, 2 mM GlutaMAX, 10 nM gastrin I, 10 mM HEPES, 1 mM N-acetylcysteine and 10 mM nicotinamide. The following niche factors were used: 50% Wnt-3A conditioned medium, 20% R-spondin-1-conditioned medium, 100ng/ml mouse recombinant noggin, 50 ng/ml human recombinant EGF, 100 ng/ml human recombinant EGF, 500 nM A83-01, and 10 µM SB 202190.	Matrigel was depolymerized using cell-recovery solution. Released cells were pelleted and mechanically digested by repeated pipetting. Cells were pelleted, supernatant was discarded, and the cells were resuspended in Matrigel and plated. Upon expansion, tumoroids were cultured without Wnt-3A, R-spondin, and Noggin.

	at 37 °C for 40 min and then further digested for 10 min by adding half the volume of TrypLE Express, and 3 mg of DNase I per sample.			
Ooft et al. (2019)	Biopsies were collected in avanced DMEM with Nutrient Mixture Ham's F-12 (Ad-DF), supplemented with 1% penicillin-streptomycin, 1% Hepes, and 1% GlutaMAX. Biopsies were stored for a maximum of 24 hours at 4°C before being dissociated with sharp needles.	Organoids were cultivated in Geltrex LDEV-free reduced growth factor basement membrane extract which was solified for 20 min at 37°C, and then overlaid with human CRC organoid medium.	Not specified, but the authors reference the 2011 paper by Sato et al., which specifies the use of advanced DMEM/F12 supplemented with penicillin-streptomycin, HEPES, Glutamax, 1× N2, 1× B27, and N-acetylcysteine, 1nM Gastrin 1, 50 ng/ml mouse recombinant EGF (varied use), 500nM A83-01 (varied use), 10 μM SB202190.	Organoids were passaged 1:5 every week. Culture medium was replaced with fresh basal culture medium. Organoids and Matrigel were mechanically disrupted using a P1000 pipette and transferred into a 15-ml Falcon tube. Further mechanical dissociation was achieved using a fire polished Pasteur pipette. Dissociated organoids were washed with 10 ml of basal culture medium and centrifuged at 200 g for 2 min. The supernatant was discarded, the pellet resuspended with Matrigel and culture medium was added.
Pasch et al. (2019)	Tissue from needle biopsy or surgical resection was placed in chelation buffer and then digested in advanced DMEM/F12 medium (Invitrogen) containing FBS, collagenase, dispase, and antibiotics. The tissues were disrupted with intermittent shaking.	Cell suspensions were maintained on ice and mixed with Matrigel at a 1:1 ratio before being plated as droplets onto multi-well culture plates and incubated at 37 °C. Plates were inverted	Feeding medium consisted of 50% ADF stuck culture medium (advanced DMEM/F12 1x, Glutamax 1x, HEPES 10mM, Penicillin/Streptomycin 100U/ml and 100mg/ml), 50% Wnt-3A conditioned medium, 50 ng/ml mouse recombinant EGF.	Medium was changed, and gels broken up by pipetting. The mixture was added to a suspension tube and placed on ice. Cells were pelleted, supernatant was discarded. Digestion could be applied at this point, before resuspending cells in culture medium and Matrigel. 1 well was

		after two to three minutes of incubation. After the mixture had solidified, cultures were overlaid with feeding medium.		typically expanded to 4-10 wells.
Yao et al. (2020)	Tumor tissues were photographed and washed in the cold PBS with penicillin-streptomycin for 5 x 5 minutes, and then minced into tiny fragments in the sterile dish on ice. Then tissue fragments were subjected to enzymatic digestion in 8 mL digestion medium containing 7 mL DMEM medium, 500 U/mL collagenase IV, 1.5 mg/mL collagenase II, 20 mg/mL hyaluronidase, 0.1 mg/mL dispase type II, 10 mM RHOK inhibitor LY27632 and 1% FBSon an orbital shaker at 37 °C for 30-60 minutes.	After digestion, tumor cells were pelleted at 300-500 g for 5 minutes and seeded into Matrigel in a well of pre-warmed 24-well flat bottom cell culture plate and overlayed with 500 mL human RCOs culture medium after incubation in a 37 degrees C and 5% CO_2 culture incubator for 5-8 minutes.	Advanced DMEM/F12 medium, 500ng/ml R-spondin 1, 100 ng/ml Noggin, 50 ng/ml EGF, 1x HEPES, 1x Glutamax, 1x Normocin, 1x Gentamicin/amphoteritin B, 1x N2, 1x B27, 1mM n-Acetylcysteine, 10 mM Niacinamide, 500 nM Alk 4/5/7 inhibitor, 3 µM p38 inhibitor, 10 nMGastrin and 10 nM Prostaglandin E2.	Tumoroids were mechanically pipetted out of Matrigel gently using cold PBS for passaging. Then organoids were resuspended in cold PBS and mechanically sheared through a pipette tip coated with 1% BSA. In contrast, dense organoids were resuspended in 12 ml TrypLE Express and mechanically sheared through 1% BSA coated pipette tip. Following these steps, organoids were washed several times with centrifugation at 200-300 g until Matrigel was cleared out. Organoid fragments were suspended in Matrigel and seeded.
Janakiraman et al. (2020)	Tumor tissue was minced with a syringe handle then cut into <1 mm3 tumor fragments. After being washed with HBSS, samples were resuspended in Ammonium–Chloride–Potassium (ACK) lysis buffer to eliminate blood cells before	1×10^6 tumor cells were mixed with 2 ml of growth factor reduced basement membrane extract (BME). Cell suspensions were seeded as 40 µl droplets into	Advanced DMEM/F12 supplemented with 10 mM HEPES, 1× GlutaMAX and 1× penicillin-streptomycin, 500 nM A83-01 ,1x B27 supplement, 50 ng/ml epidermal growth factor, 10 nM gastrin, 1 mM N-acetyl-L-cysteine, 10 mM nicotinamide, 10 nM prostaglandin E2, 6	PDTOs were passaged every 7 days, not further specified.

	being digested by 0.2 μ/ml of Liberase DH containing 10 μm Y-27632 for 60 min at 37 degrees C. Cell suspension was filtered through a250 μm sieve followed by a 100 μm cell strainer. Cell suspension was collected, spun down and resuspended in basal culture medium: advanced DMEM/F12 supplemented with 10 mM HEPES, 1× GlutaMAX and 1× penicillin-streptomycin.	a prewarmed six-well plate with seven droplets per well and incubated at 37° C for 10 min to solidify BME. Three milliliters of complete organoid culture medium was added to each well and maintained at 37°C with 5% CO_2.	μM SB20219 and 10 μM Y27632.	
Narashiman et al. (2020)	Tumor samples were minced and enzymatically digested in organoid digestion medium which consisted of DMEM containing Collagenase IV 67.5 U/mL, Dispase 0.23 U/mL, Hyaluronidase 8–20 U/mL, DNase Type I 50 Kunitz units/mL, 100 U/mL penicillin, and 100 μg/mL streptomycin in a water bath at 37°C for 30–60 minutes.	Not specified.	Advanced DMEM/F12, 10 mmol/L Hepes, 1× glutamax, 10 mg/L gentamicin, 1× antibiotic-antimycotic, 2× B27, 500 nmol/L A83–01, 50 ng/mL hEGF, 1 nmol/L Gastrin 1 human, 1 mmol/L N-Acetyl-L-cysteine, 5 μmol/L SB202190, 10 μmol/L SB431542, and 10 μmol/L Y27632.	Organoids were passaged upon reaching 100–200 μm in diameter by digestion with TrypLE (Gibco) at 37°C followed by tituration with a pipette. Cells were replated in Matrigel, with the general aim to expand the number of wells by at least double at each passage.
Xu et al. (2021)	Biopsies from mCRC patients were collected in cold PBS for a maximum of 24 hours at 4°C before being minced, and subsequently digested in 5ml enzyme buffer for 60 minutes at 37°C while shaking. The	Cells were resuspended in culture medium and $5x10^3$ cells/25 μl were dispensed into HA-Coll sponges (produced and supplied by PISHON	1x advanced DMEM/F12, 1x GlutaMAX-1, 1x HEPES, 1x B27, 1x N2, 50 ng/ml EGF, 100ng/ml Noggin, 500 ng/ml R-spondin 1, 10nM Gastrin, 10 μM Y-27632, 10 mM nicotinamide, 1 mM N-acetylcysteine,	Sponges were mechanically torn by tweezers to release the tumor organoids and organoids were collected in a tube and centrifuged at 400g for 5min at 4°C to remove the medium. 1ml TrypLE Express

	composition of digestion enzyme buffer was Collagenase type IV (2mg/ml), Hyaluronidase (200 µg/ml), DNase I (200 µg/ml) and Y-27632 (10 µM). For thorough digestion, the remaining fragments were additionally dissociated with TrypLE Express at 37°C for an additional 15 min. Tissue fragments were discarded through 70 µm Nylon Cell Strainer and the dissociated cells were collected and centrifuged at 400g for 5min at 4°C.)	Biomedical, Taiwan) which were pre-plated at 48-well flat bottom cell culture plates. After 4 hours' incubation in a 37°C, 5% CO2 cell culture incubator, an extra 250µl culture medium was added into wells to submerge sponges.<br><br>Matrigel cultures were established using the same protocol as Vlachiogannis et al.	500 nM A83-01, 10 µM SB202190.	containing 1 mM EDTA was added and PDOs were incubated at 37°C for 5-10min, after then, The PDOs were then harvested out by mechanically pipetting using 200µl yellow pellet tips. The isolated PDO fragments were then washed with PBS, pelleted, resuspended in culture medium and re-seeded at an appropriate ratio of 1:2 to 1:5.<br><br>PDTOs in matrigel were passaged using the same protocol as Vlachiogannis et al.
Wang et al. (2021)	Tumor tissue was washed with 10 mL Hank's Balanced Salt Solution (HBSS) containing antibiotics, minced with scissors, and digested in 5 mL of 5 mg/mL collagenase type II in DMEM/F12 for approximately 4 h at 37°C with gentle shaking and intermittent pipetting. The digested tissue suspension was strained with a 70-µm filter. The strained suspension was centrifuged at 300g, and red blood cells were lysed using lysis buffer for 5 min.	Counted cells were resuspended in Matrigel basement membrane matrix. Next, 30-µL drops of the Matrigel cell suspension were allowed to solidify on prewarmed 60-mm culture plates in a 37°C and 5% CO_2 cell culture incubator for 30 min.	Organoids were cultured in advanced DMEM/F12, not further specified.	Not specified.

	Dissociated cells were washed and counted.			
Ooft et al. (2021)	Biopsies were transferred to a sterile 15-ml tube containing advanced DMEM with Nutrient Mixture F-12 Hams (Ad-DF), supplemented with 1% penicillin-streptomycin, 1% HEPES, and 1% GlutaMAX (referred to as Ad-DF+++). Tubes containing fresh biopsies were kept at 4°C and processed within 24 h. Biopsies were mechanically dissociated with needles to cell clumps and washed with Ad-DF+++.	Organoids were cultivated in Geltrex LDEV-free reduced growth factor basement membrane extract which was solified for 20 min at 37°C, and then overlaid with human CRC organoid medium	Ad-DF+++, 10% Noggin-conditioned medium, 20% R-spondin1-conditioned medium, 1x B27 supplement without vitamin A, 1.25 mM N-Acetylcysteine, 10 mM nicotinamide, 50 ng/mL human recombinant EGF, 500 nM A83-01, 3 µM SB202190 and 10 nM prostaglandin E2.	Organoids were passaged approximately every week by incubating in TrypLE Express for 5-10 min at 37℃ to dissociate organoids to single cells and replating in fresh Geltrex. After passaging, 10 µM Y-27632 was added to CRC-medium for the first 2-3 days.
Park et al. (2021)	Both tumor and adjacent normal tissues were collected. Tumor tissue was incubated with collagenase type II, dispase type II, and Y-27632 for 30 min at 37°C to dissociate the cells.	Cancer cells were washed with PBS and centrifuged at 300g for 3 min at room temperature. The cells were then embedded in Matrigel on ice (growth factor reduced, phenol red free) and seeded in 24-well plates, followed by addition of culture medium.	1× B27, 1.25 mM N-acetyl cysteine, 50 ng/ml human epidermal growth factor, 50 ng/ml human Noggin, 10 nM gastrin, 500 nM A83-01 and 100 mg/ml primocin. To prevent anoikis, 10 µM Y-27632 was added to the culture medium for the first 2-3 days.	When organoids were >200 µm in diameter, they were passaged by pipetting using Gentle Cell dissociation reagent, according to manufacturer's instruction.
Cui et al. (2022)	Tumor tissue was washed five times with cold PBS supplemented with antibiotics, and then minced into fragments	The supernatant from the digestion step was passed through a 70-µm cell strainer, centrifuged, and	DMEM/F12 supplemented with 1X B27, Glutamax, 10 mM HEPES, 100 µg/mL primocin, 50 ng/mL recombinant human	Not specified.

	with a size smaller than 2 mm^3. The tissue fragments were chemically digested with 1 mg/mL collagenase A at 37°C for 10–15 min on an orbital shaker, and then mechanically dissociated by repetitive pipetting in cold DMEM/F12 4–5 times.	seeded into 100% matrigel in a 24-well tissue-culture plate. After polymerization for 10 min at 37°C, the matrigel dome was overlaid with 500 µL of organoid culture medium.<br><br>For drug screening, they used a nested array chip plate modelled after a standard 96-well plate, with a 3D-implanting hole in the center of each well measuring 2.5 mm in diameter and 1.5 mm in height, where matrigel with cancer cells were deposited.	EGF, 10 nM gastrin, 500 nM A83-01, 1.25 mM N-acetylcysteine, 10 mM nicotinamide, 100 ng/mL recombinant human Noggin/10% Noggin conditioned media, and 20% R-spondin1 conditioned media. 10 µM Y-27632 dihydrochloride kinase inhibitor was also added to the culture medium for 2–3 days.	
Cho et al. (2022)	Fresh tumor tissue was washed with PBS and minced using a gentleMACS Dissociator. Processed samples were passed through a 70-µm cell strainer to eliminate macroscopic tissue debris and then washed with basal medium consisting of advanced DMEM/F12 supplemented with penicillin-streptomycin, 10 mM	Dissociated tumor cell pellets were seeded with Matrigel in 24-well plates. After the Matrigel had solidified, organoid medium was added.	Organoid medium consisted of advanced DMEM/F12, supplemented with penicillin-streptomycin, 10 mm HEPES, and GlutaMAX, 10% R-spondin1 conditioned medium, 10% Noggin conditioned medium or 100 ng·mL−1 recombinant Noggin, 50 ng·mL−1 recombinant human EGF, B27, 1.25 mm N-acetyl cysteine, 10 mm nicotinamide, 3 µm	Organoids embedded in Matrigel were collected and dissociated by mechanical disruption. Subsequently, the remaining Matrigel was washed out with cold PBS and the cells were reseeded with fresh Matrigel. To prepare single-cell suspensions, organoids were dissociated using TrypLE Express before reseeding.

	HEPES, and GlutaMAX.		SB202190, 500 nm A83-01, 10 nm prostaglandin E2, 10 nm gastrin, and 100 µg/mL1 Primocin.	
Ding et al. (2022)	Tumor were kept in transfer media (not specified) and on ice after dissection. Tissue was minced before mixing with 10 ml of an enzymatic solution consisting of 3 mM CaCl2, 1 mg/ml Collagenase, 0.1 mg/ml DNase I, 10 µM Y-27632, and 100 ug/ml primocin. Minced tissue samples were dissociated with gentle agitation in enzymatic solution for 30 minutes at 37°C before quality check. If large cell clumps were observed, an additional 15-20 minutes of digestion was performed until the tissue was mostly dissociated into single cells. After digestion, cells were filtered through a 70 µM cell strainer, and yield and cell viability were determined by a Countess II cell counter.	Suspended cells from primary tissue was added to Matrigel, followed by mixing with a biphasic liquid (oil) to generate microfluidic-based droplet micro-organospheres (MOS) in a controlled manner. The generated MOS are demulsified to remove excess oil and then cultured as suspension droplets in organoid medium in 6-well lo binding plates.	1x DMEM/F12, 10mM HEPES, 1x Glutamax, 100U/ml penicillin/streptomycin, 1x Primocin, 1x B27, 1.25 nM N-acetylcysteine, 5 mM Nicotinamide, 100 ng/ml Noggin, 500 nM SB202190, 50 ng/ml EGF, 1x B27 minus vitamin A, 1x N2.	Not specified.
Hsu et al. (2022)	Tissues were stripped of underlying muscle layers and submucosa with surgical scissors and chopped into ~1-mm pieces in cold PBS-DTT (PBS with	Tumor cell pellets were resuspended in Matrigel and plated onto 24-well culture plates (40 µL droplets	Advanced DMEM/F12 medium plus 1 mmol/L GlutaMAX, 1 mmol/L HEPES, and 100 U/mL penicillin/streptomycin, 10% Noggin conditioned medium, 10	Organoid-Matrigel mixtures were collected into 15-mL tubes and incubated with Cell Recovery Solution on ice for 40 to 60 minutes to dissolve Matrigel.

	10 mmol/L dithiothreitol). The supernatant was removed after centrifuging at 300g for 5 minutes, and then a digestion buffer was added, consisting of advanced DMEM/F12 medium with 2% FBS, 100 U/mL penicillin/streptomycin, 500 U/mL collagenase, and 125 µg/mL Dispase type II, for 30 minutes at 37°C, shaking every 5 minutes. Tumor samples were washed with 10 mL of ADF-FBS medium and spun at 300g for 5 minutes.	pr. well), and solidified before culture medium was added and the organoids were incubated at 37°C.	nmol/L gastrin I, 500 nmol/L A83–01, 10 mmol/L SB202190, 10 mmol/L nicotinamide, 1X B27 supplement, 1X N2 supplement, 1 mmol/L N-acetyl cysteine, and 50 ng/mL human recombinant EGF.	Released organoids were spun down at 60g for 5 minutes, resuspended in 1 to 2 mL of PBS, and broken down into small fragments by physical pipetting 30x with a p1000 pipette. In some cases, organoids were further dissociated with TrypLE Express Solution at 37°C for 3 minutes to obtain smaller fragments or homogeneous single cells. Resulting fragments and cells were washed with 10 mL of ADF medium, pelleted at 300 × g for 5 minutes, resuspended with Matrigel and plated into new 24-well plates with a typical split ratio = 1:3-1:4.
Mo et al. (2022)	Tissues were washed in ice-cold PBS with penicillin/streptomycin for 3 x 5 min, and cut into 1-3 mm^2 pieces in the sterile dish on ice. Then tissue fragments were washed in ice - cold PBS with penicillin-streptomycin for 3 x 5 min and digested in 10 mL digestion medium containing 10 mL DMEM medium, 500 U/mL collagenase IV, 1.5 mg/mL collagenase II, 20 mg/mL hyaluronidase, 0.1 mg/mL Dispase type II, 10 µm RHOK	Isolated cells were embedded in Matrigel in a well of pre-warmed 24-well flat bottom cell culture plate. After the Matrigel balls were polymerized, 500 µL of culture medium was added.	Culture medium consisted of 1x Advanced DMEM/F12, 1x HEPES, 1x Glutamax, 1x Penicillin/streptomycin, 1x N2, 1x B27, 50 ng/mL EGF, 1mM N-acetylcysteine, 10mM Nicotinamide, 1x Normocin, 1x gentamicin-amphotericin B, 500 nM A83-01, 10 nM Prostaglandin E2, 10 nM Gastrin, 3 µM SB202190, 500 ng/mL R-spondin 1, 100 ng/mL Noggin.	Matrigel containing organoids were pipetted into 15 mL centrifuge tube using ice-cold PBS and washed with centrifugation at 200g. The suspension was removed, and the pellets were resuspended in ice-cold PBS with pipetting 30-60 times using a 1 mL pipette. The cell mixture was washed and embedded in Matrigel at a 1:2 ratio.

	inhibitor Y-27632 and 1% fetal bovine serum on an orbital shaker for 30 min at 37°C. The suspension was collected, filtered through a 100 μm cell filter and then centrifuged at 200g for 5 min.			
Lv et al. (2022)	Tumor tissue was photographed and washed in the cold PBS with penicillin-streptomycin for 5 x 5 minutes, and then minced into tiny fragments in the sterile dish on ice. Tissue fragments were then subjected to enzymatic digestion in 8 mL digestion medium containing 7 mL DMEM medium, 500 U/mL collagenase IV, 1.5 mg/mL collagenase II, 20 mg/mL hyaluronidase, 0.1 mg/mL Dispase type II, 10 mM RHOK inhibitor ly27632 and 1% fetal bovine serum on an orbital shaker at 37 degrees C for 30-60 minutes.	After digestion, tumor cells were pelleted at 300-500g for 5 minutes and seeded into Matrigel in a well of pre-warmed 24-well flat bottom cell culture plate and overlayed with 500 mL human RCOs culture medium after incubation in a 37 degrees C and 5% CO2 culture incubator for 5-8 minutes.	Advanced DMEM/F12 medium, 500ng/ml R-spondin 1, 100 ng/ml Noggin, 50 ng/ml EGF, 1x HEPES, 1x Glutamax, 1x Normocin, 1x gentamicin-amphoteritin B, 1x N2, 1x B27, 1mM n-Acetylcysteine, 10 mM Niacinamide, 500 nM Alk 4/5/7 inhibitor, 3 μM p38 inhibitor, 10 nM Gastrin and 10 nM Prostaglandin E2.	Tumoroids were mechanically pipetted out of Matrigel gently using cold PBS for passaging. Then organoids were resuspended in cold PBS and mechanically sheared through 1% BSA coated pipette tip. In contrast, dense organoids were resuspended in 1-2ml TrypLE Express and mechanically sheared through 1% BSA coated pipette tip. Following these steps, organoids were washed several times with centrifugation at 200-300g until Matrigel was cleared out. Organoids fragments were resuspended in Matrigel and seeded.
Tang et al. (2023)	Tumor samples were washed with 10 mL of HBSS containing 5% antibiotics 8–10 times, and minced with scissors to 1 mm^3 in size. The minced tissue was digested in 5 mL of 5 mg/mL collagenase type II in	Tumor cells were washed, counted, and resuspended in a mixture of Matrigel basement membrane Matrix and organoid culture medium. Then, 30 mL	Not specified, but the authors reference the 2011 paper by Sato et al., which specifies the use of Advanced Dulbecco's modified Eagle medium/F12 supplemented with penicillin/streptomycin,	Not specified.

	DMEM/F12 for approximately 3 h at 37°C on a shaker. After filtering, the suspension was centrifuged at 1200 3 g for 2 minutes, and red blood cells (RBCs) were removed by adding RBC lysis buffer for 5 minutes.	drops of the Matrigel cell suspension were incubated in a 37°C and 5% CO2 cell culture incubator for 10 min to solidify before culture medium was added to each plate. The cells were incubated in a 37°C and 5% CO2 cell culture incubator.	10 mmol/L HEPES, Glutamax, 1× N2, 1× B27, 1 mmol/L N-acetylcysteine, EGF (varied use), A83-01 (varied use), and SB202190 (varied use) for colon cancer organoids.	
Martini et al. (2023)	Tumor tissue was collected from surgical specimens or biopsy, transferred into sterile PBS, and processed within 24 h. After a 1600 rpm centrifugation, pellets were collected and transferred to a plate with a sterile scalpel, then transferred into a tube with medium enriched with antibiotics, DNAse and collagenase for tissue dissociation and then incubated for 15 min at 37°C, resuspended and filtered through a 100-μm cell strainer.	The resultant cells were cultured in low-attachment wells with culture medium. After 3-5 days, clumps were dissociated in TrypLE for 5-10 min, counted and cultured in Matrigel at 37°C, 5% CO_2.	Wnt-conditioned medium, R-spondin 1 conditioned medium, Noggin conditioned medium, B27, N2, N-acetylcysteine, Nicotinamide, human EGF, Gastrin 1, Prostaglandine E2, A83-01, SB202190, Primocin. Upon dissociation of cells or passage, Y-27632 was added to the growth medium.	Matrigel drops are gently disrupted by pipetting or trypsinized with TrypLE Express, reseeded into matrigel drops, and covered with medium.
Wang et al. (2023)	Samples were collected in RPMI 1690 supplemented with 5% penicillin–streptomycin and stored for a maximum of 10 hours at 4°C. Then the tumor tissue	Tumor cells were collected, washed, counted, and resuspended in Matrigel basement membrane matrix, which was	Advanced DMEM/F12 containing N-acetylcysteine, EGF, FGF-10, FGF-basic, Y-27632, A-83-01, SB202190, nicotinamide, Noggin, R-spondin, Wnt3a,	Not specified.

was washed in HBSS containing antibiotics, minced into tiny pieces, and digested with 5 mL DMEM/F12 containing collagenase type II on a shaker for approximately 4 hours at 37°C. To eliminate erythrocytes, the digested tissue suspension was incubated with lysis buffer and centrifuged at 300g for 5 minutes.	dispended as 30-µL drops into prewarmed 60-mm culture plates and allowed to solidify for 30 minutes at 37°C. The cells were then overlaid with organoid culture medium and incubated at 37°C and 5% CO2.	HEPES, GlutaMAX, B27, and N2.

Supplementary Table S6. Point estimates and 95% confidence intervals for accuracy, sensitivity, specificity, positive predictive value (PPV), and negative predictive value (NPV) of studies included in the meta-analysis.

Study	Accuracy	Sensitivity	Specificity	PPV	NPV
Vlachiogannis et al.	0.95 (0.71 – 1.00)	1.00	0.93 (0.57 – 1.00)	0.88 (0.38 – 1.00)	1.00
Ooft et al.	0.91 (0.64 – 0.95)	1.00	0.82 (0.36 – 0.91)	0.85 (0.46 – 0.92)	1.00
Yao et al.	0.85 (0.74 – 0.91)	0.92 (0.76 – 0.97)	0.79 (0.63 – 0.88)	0.79 (0.63 – 0.88)	0.92 (0.76 – 0.97)
Wang et al.	0.80 (0.67 – 0.88)	0.63 (0.43 – 0.77)	0.94 (0.76 – 0.97)	0.91 (0.62 – 0.95)	0.74 (0.58 – 0.84
Ding et al.	0.75 (0.13 – 0.88)	0.60 (0.00 –0.80)	1.00	1.00	0.60 (0.00 – 0.80)
Lv et al., irinotecan	0.74 (0.61 – 0.81)	0.72 (0.55 – 0.84)	0.76 (0.59 – 0.85)	0.74 (0.55 – 0.84)	0.74 (0.57 – 0.83)
Lv et al., radiation	0.71 (0.59 – 0.79)	0.94 (0.65 – 1.00)	0.65 (0.51 – 0.75)	0.46 (0.24 – 0.55)	0.65 (0.56 – 0.67)
Lv et al., 5-FU	0.66 (0.54 – 0.75)	0.74 (0.48 – 0.87)	0.63 (0.47 – 0.74)	0.45 (0.26 – 0.58)	0.86 (0.69-0.93)
Tang et al.	0.75 (0.67 – 0.81)	0.75 (0.62 – 0.83)	0.75 (0.62 – 0.82)	0.72 (0.60 – 0.81)	0.78 (0.66 – 0.86)
